# 1780. Analysis of Appropriate Outpatient Prescriptions based on Diagnosis Codes, Tennessee, 2020

**DOI:** 10.1093/ofid/ofac492.1410

**Published:** 2022-12-15

**Authors:** Youssoufou Ouedraogo, Christopher D Evans, Christopher Wilson, Milner B Staub, Sophie E Katz

**Affiliations:** Tennessee Department of Health, Nashville, Tennessee; Tennessee Department of Health, Nashville, Tennessee; Tennessee Department of Health, Nashville, Tennessee; Vanderbilt University Medical Center, Nashville, Tennessee; Vanderbilt University Medical Center, Nashville, Tennessee

## Abstract

**Background:**

In the US, approximately 60% of antimicrobial prescriptions (Rx) are written in the outpatient setting, and roughly 30% of those are unnecessary. The Tennessee Department of Health (TDH) previously described outpatient antimicrobial use in 2016 and 2018. TDH acquired additional data from the IQVIA Longitudinal Prescription and Medical Claims datasets. We sought to link antimicrobial Rx to diagnosis claims from preceding outpatient office visits to measure appropriateness of statewide antimicrobial Rx.

**Methods:**

IQVIA medical claims data and antimicrobial Rx data for calendar year 2020 were used in this study. A unique patient ID was used to track antimicrobial Rx over time. Prescriptions filled within 7 days after a patient’s medical visit were included. Diagnoses codes from the medical visits were categorized as Tier 1 (diagnoses that always require antimicrobial therapy) and Tier 3 (diagnoses that never require antimicrobial therapy). Tier 2 diagnoses were excluded. We compared Tier 1 prescriptions versus Tier 3 Rx volume.

**Results:**

We identified 2.3 million Rx linked to a preceding outpatient office visit within 7 days; 70.5% were filled within 3 days of the corresponding office visit, 14.1% within 4–5 days, and 15.4% within 6–7 days. Of all included Rx, 16.4% were prescribed following a pure Tier 1 diagnosis, and 83.6% were prescribed following a pure Tier 3 diagnosis. Diagnoses for respiratory conditions accounted for 38.4% of the pure Tier 3 visits, urinary tract conditions for 17.7%, gastrointestinal conditions for 16.2%, and skin and soft tissue conditions for 15.8%. For the respiratory conditions, viral upper respiratory infection accounted for the highest percent of antimicrobials prescribed (27.1%), followed by asthma/allergy indications (17%), and bronchitis (8.9%).

**Conclusion:**

A significant number of antimicrobials prescribed in Tennessee in 2020 were able to be linked to office visit diagnosis codes. Most Rx were prescribed for indications that do not require antimicrobial therapy. Respiratory conditions accounted for the highest percentage of visits resulting in antimicrobial therapy that did not require treatment. Continued educational efforts and provider feedback of these data are planned to combat unnecessary overprescribing in Tennessee.

**Disclosures:**

**Milner B. Staub, MD, MPH**, Gilead: Stocks/Bonds|Johnson & Johnson: Stocks/Bonds.

